# Comparison of epidural dexmedetomidine to fentanyl in reducing ropivacaine dose in Programmed Intermittent Epidural Bolus plus Patient Controlled Epidural Analgesia during labor: A randomized, double-blind, controlled study

**DOI:** 10.3389/fmed.2022.935643

**Published:** 2022-10-17

**Authors:** Ru-Ying Pang, Yao-Hua Shen, Xiao-Qin Jin, Hai-Feng Xu, Yang Wang, Bin-Xiang Zhu, Su-Feng Lin, Fei Xiao

**Affiliations:** ^1^Department of Anesthesia, Hangzhou City Lin-Ping District Women and Children Care Hospital, Hangzhou, China; ^2^Department of Anesthesia, Jiaxing Women and Children’s Hospital of Wenzhou Medical University, Jiaxing, China; ^3^Jiaxing University Affiliated Women and Children Hospital, Jiaxing, China

**Keywords:** dexmedetomidine, fentanyl, labor analgesia, epidural, ropivacaine

## Abstract

**Background:**

Dexmedetomidine has been documented to reduce the dose of both intrathecal local anesthetic during cesarean delivery, and the concentration of ropivacaine needed for inducing analgesia during labor. However, few studies have compared adjuvant dexmedetomidine to fentanyl on how they impact the dose of ropivacaine required during labor. The aim of the current study was to evaluate the efficacy of epidural dexmedetomidine at doses of 0.3, 0.4, or 0.5 and 2 μg/ml of fentanyl (the traditional clinical concentration), when added to epidural 0.125% ropivacaine.

**Methods:**

This was a randomized, double-blinded study that comprised one hundred eighty-eight patients, allocated into four groups receiving either epidural fentanyl at 2 μg/ml, or dexmedetomidine at 0.3, 0.4, or 0.5 μg/ml for labor analgesia. The primary outcome was the amount of ropivacaine necessary per hour. Secondary outcomes included visual analogue pain scale (VAS), motor block (Bromage Scale), side effects, patient satisfaction, and neonatal outcomes.

**Results:**

At the completion of the study, data from 165 participants were analyzed. The mean hourly amount of epidural ropivacaine administered was 16.2 ± 3.3, 14.0 ± 3.1, 13.1 ± 3.7 and 12.1 ± 2.5 ml/h in the 2 μg/ml fentanyl group, and the 0.3, 0.4 and 0.5 μg/ml dexmedetomidine groups, respectively. There was a significant difference among groups in the mean hourly consumption of epidural ropivacaine (*P* < 0.0001 for 1 way ANOVA). The frequency of PCEA (patient-controlled epidural analgesia) was significantly higher in the fentanyl group than in the three dexmedetomidine groups (*P* < 0.001), and similar among the dexmedetomidine groups. The mean values of the VAS among all groups were similar over time, *P* > 0.05. The incidence of pruritus in the fentanyl group was 17.5%, whereas no patient experienced pruritus in any of the dexmedetomidine groups, *P* < 0.0001.

**Conclusion:**

The study demonstrated that epidural dexmedetomidine (0.3 and 0.4 μg/ml) was superior to standard dose epidural fentanyl in reducing the mean hourly amount of ropivacaine administered, and minimizing opioid-related side effects. Further large and multicenter studies would be necessary to confirm the benefits of dexmedetomidine, and potentially serve as an alternative to opioids for routine use in labor analgesia.

**Clinical trial registration:**

[http://www.chictr.org.cn/showproj.aspx?proj=62846], identifier [ChiCTR2000039067].

## Introduction

Opioids are usually used in epidural labor analgesia to reduce the dose requirements of epidural local anesthetic agents, specifically intended to minimize the side effect of an epidural blockade, including maternal motor block and hypotension. However, epidural opioids are also associated with side effects, such as pruritus and reduced fetal heart rate variability (1). Therefore, non-opioid local analgesic adjuvants have been studied as a mean to reduce the quantity of epidural local anesthetic agents. The α_2_ receptor agonist, dexmedetomidine, has been shown to provide opioid-sparing analgesia when administered peripheral, epidural, or intrathecal as an adjuvant (2–8).

In a prior study, dexmedetomidine decreased the EC_50_ (median effective concentration) of ropivacaine for inducing epidural labor analgesia at an optimal dose of 0.4 μg/ml (6). Nevertheless, there have been few studies that have assessed the efficacy of epidural dexmedetomidine as a local anesthetic adjuvant for labor analgesia via the model of Programmed Intermittent Epidural Bolus (PIEB) plus Patient Controlled Epidural Analgesia (PCEA).

The primary aim of the current study was to evaluate the efficacy of epidural dexmedetomidine at three different doses: 0.3, 0.4, or 0.5 μg/ml, compared to fentanyl at the traditional clinical concentration of 2 μg/ml, as adjuvants to epidural 0.125% ropivacaine. The secondary aims were to define the dose-response of epidural dexmedetomidine at doses ranging from 0.3 to 0.5 μg/ml, and assess the visual analogue pain scale (VAS), motor blockade (Bromage Scale), medication side effects, patient satisfaction, and neonatal outcomes. We therefore hypothesized that dexmedetomidine used as an analgesia adjuvant could reduce the mean hourly dose requirement of ropivacaine for PIEB plus PCEA during labor when compared to the combination of ropivacaine with fentanyl.

## Methods

### Design

The study was registered in the Chinese Clinical Trial Registry on 1st February, 2021 (registry number ChiCTR2000039067) prior to enrollment of the first patient (February 3, 2021); Ethical approval for this study (Ethical Committee No. LLSC-KYKT-2022-0002-A) was provided by the Ethical Committee of Lin-ping District Women and Children Care Hospital, Hangzhou, China (Chairperson Prof. Shen Yuejian) on January 17, 2021. Written, signed informed consent was obtained from all study subjects. This was a randomized, double-blinded study to assess the mean hourly requirement of ropivacaine administered, comparing the use of adjuvant fentanyl in standard doses to adjuvant dexmedetomidine in three different concentrations.

### Subjects and setting

Recruited for this study were nulliparous parturients with healthy singleton pregnancies, gestational age ≥ 37 weeks, American Society of Anesthesiologists Physical Status II spontaneous onset of labor, latent phase of labor with cervical dilation of 2–5 cm, and painful contractions requiring labor epidural analgesia. Patients with preeclampsia or hypertension, preexisting or gestational diabetes, BMI > 35 kg/m^2^, contraindications to local anesthetics, dexmedetomidine, and fentanyl were excluded from this study. We enrolled 240 patients for initial eligibility assessment with a goal of 33 patients for each group for final analysis.

### Study protocol

Patients were randomized into four groups to receive four adjuvant medications: 2 μg/ml fentanyl, or 0.3, 0.4, or 0.5 μg/ml dexmedetomidine (Dexmedetomidine Hydrochloride Injection, 2 ml: 200 μg, Yangtze River Pharmaceutical Group Co., Ltd., Jiangsu, China; preservative-free and contains no additives or chemical stabilizers), with the randomization scheme generated by FX. He was not involved in any clinical patient management, but did collect the study data, using Microsoft Excel (Microsoft Corporation, Redmond, WA, USA). The randomization scheme was kept in sequentially numbered opaque envelopes and opened after the first patient was enrolled. The study drugs were prepared in sterile conditions by an anesthesia assistant who had no involvement in clinical patient management. All study participants were blinded to their group assignment and the study drug administered.

Following parturient arrival in the labor room, a peripheral venous catheter was inserted and routine monitoring initiated (blood pressure cuff, pulse oximeter, electrocardiography leads, respiratory rate monitor, and fetal heart rate monitor). When the patient required labor analgesia, an epidural catheter (two holes, 19G; Shanghai SA Medical Technology Co., Ltd., Shanghai, China) was placed at the L3-4 interspace inserting 3–4 cm into the epidural space by 1 of 3 attending anesthesiologists (R-YP, Y-HS, and X-QJ). As a test dose, a combination of 45 mg lidocaine and 15 μg epinephrine, was injected through the catheter.

After a satisfactory test dose, the patient received 10–13 ml of study solution as an epidural bolus to relieve the labor pain. If the patient reported a visual analogue pain scale (9) (VAS) value > 3 on a 0–10 scale (0, no pain; 10, worst imaginable pain) 20 min following the epidural bolus, the patient was excluded from this study because the epidural catheter was regarded as an “unreliable” catheter (subsequently to be replaced or managed by the anesthesiologist).

Forty-five minutes following injection of the initial epidural study bolus, a PIEB plus PCEA infusion protocol was initiated using an Apon infusion pump (Jiangsu Apon Medical Technology Co., Ltd., Jiangsu, China) according to the following parameters: PIEB analgesia was initiated with an 8 ml bolus and maintained using a programmed bolus of 8 ml at 40 min intervals; additionally, 8 ml PCEA boluses were available for supplementation with a 15 min lockout interval, and maximum dose of 30 ml/h. Patients experiencing “breakthrough pain” were treated with a bolus of 10 ml of 0.25% ropivacaine plus 100 μg of fentanyl according to our institutional practice. If the patient still reported a VAS > 3 after a bolus or required yet an additional bolus in 1 h, she was excluded from the study.

The primary outcome of this study was the mean amount of ropivacaine administered per hour, which was defined as the total amount administered (consumption) of 0.125% ropivacaine volume divided by the infusion duration. Secondary outcomes were also studied as follows: the number of PCEA boluses the patient used; the values of VAS recorded at the following time-points: prior to epidural catheter placement, 20 min following the initial induction bolus, and subsequently at 2 h intervals until delivery; the motor block level as assessed according to the Bromage scale (10) (0–3, 0 = ability to move all joints in the leg, 1 = able to bend the knees and ankles, 2 = only able to move the ankle, and 3 = not able to move any leg joint) 20 min after the initial induction bolus and then at 2 h intervals until delivery; the incidence of side effects including hypotension (a decrement > 20% from baseline blood pressure, or an absolute value <90 mm Hg; if hypotension occurred, the patient’s position was changed to left lateral and the blood pressure was checked again; if hypotension persisted, ephedrine 5 mg was given intravenously and repeated as required), bradycardia (heart rate < 50 bpm, rescued by atropine 0.5 mg), pruritus, maternal sedation (none [awake and alert], mild [awake but drowsy], moderate [asleep but arousable], and severe [not arousable]), respiratory depression (oxygen saturation < 90%), nausea and vomiting, and shivering. Newborn umbilical artery pH, Apgar score at 1 and 5 min, and delivery mode (vaginal vs. cesarean delivery) were also recorded and analyzed. Patient satisfaction was also assessed using a 1–5 verbal score (1 = not satisfied at all, 5 = extremely satisfied).

### Sample size

According to PASS (version 11.0.7; NCSS, LLC, Kaysville, UT, USA) software and prior studies, to detect a clinically meaningful difference of 20% in hourly ropivacaine consumption among groups (α = 0.05 and 1−β = 0.8), thirty-five subjects would be needed for each group (11). In order to account for attrition, the Institutional Review Board (IRB) agreed to the recruitment of 60 patients for each group.

### Statistical analysis

The distribution of univariate data was assessed via the Kolmogorov–Smirnov test. Normal distribution data such as the demographic data, the total hourly ropivacaine consumption, and pain scores were presented as Mean ± SD and analyzed via one-way analysis, and the Tukey’s multiple comparisons test was used for pairwise comparison. Non-normal distribution data was presented as Median (range) and tested with the Kruskal–Wallis test, and the *post hoc* Dunn’s test was applied to analyze the pairwise comparison. Categorical trend data such as incidence of side effects and Bromage score were analyzed using the Cochran–Armitage chi-square test for trend, if an overall test of difference among groups was significant, chi-square tests were used for pairwise comparison. *P* < 0.05 was considered significant. Where Bonferroni corrections were applied, adjusted *P* values are presented. Analyses were performed using IBM SPSS Statistics for Windows version 22.0 (IBM Corp, Armonk, NY, USA) and GraphPad Prism version 5.0 (GraphPad Software Inc., San Diego, CA, USA).

## Results

**FIGURE 1:**
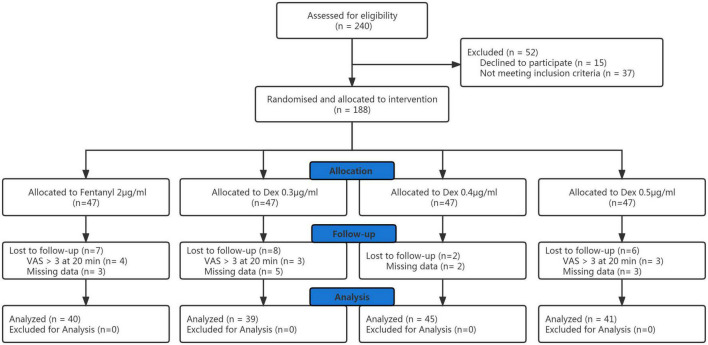
CONSORT diagram.

Of the initial 240 participants enrolled with written informed consent, data from 165 participants were involved in the final analysis (Figure 1). Patient demographic data are shown in Table 1 and there was no significant difference among groups. There were no significant differences among the groups in the progress of labor, neonatal outcomes. Totally, there were 11 patients who underwent cesarean delivery in the four groups. Two patients in 0.4 and 0.5 μg/ml dexmedetomidine groups because of fetal distress, and a patient in 0.4 μg/ml dexmedetomidine group underwent cesarean delivery because of fetal macrosomia. Eight patients in the four groups underwent cesarean delivery because of cephalopelvic disproportion. There was no significant difference in the cesarean delivery rate among groups.

**TABLE 1:** Demographics, labor characteristics, and neonatal outcomes of laboring patients.

	Fentanyl 2 μg/ml	Dexmedetomidine 0.3 μg/ml	Dexmedetomidine 0.4 μg/ml	Dexmedetomidine 0.5 μg/ml
Sample size, *n*	40	39	45	41
Age, years	27.6 ± 3.7	28.3 ± 3.7	27.4 ± 3.4	28.8 ± 3.5
BMI, kg/m^2^	26.1 ± 3.0	27.5 ± 2.4	27.0 ± 3.2	27.2 ± 2.4
Gestational age, weeks	39.3 ± 1.1	39.3 ± 1.0	39.1 ± 1.0	39.5 ± 2.0
Cervical dilation at epidural placement, cm	2.1 ± 0.3	2.2 ± 0.4	2.2 ± 0.4	2.1 ± 0.4
Epidural analgesia to cervix complete, min	240 (160, 344)	195 (115, 316)	240 (156, 421)	220 (145, 300)
Epidural analgesia to delivery, min	293 (226, 401)	255 (176, 377)	307 (193, 502)	278 (188, 403)
Cesarean delivery, *n* **(%)**	2 (5.0)	2 (5.1)	4 (8.9)	5 (12.2)
Patient satisfaction score, 1–5	4.3 ± 0.8*	4.8 ± 0.4	4.7 ± 0.6	4.8 ± 0.5
Neonatal weight, g	3333 ± 373	3366 ± 425	3285 ± 433	3339 ± 408
1-min Apgar score	9.8 ± 0.5	9.7 ± 0.7	9.8 ± 0.5	9.7 ± 0.6
5-min Apgar score	9.9 ± 0.4	9.9 ± 0.2	10 ± 0.0	9.9 ± 0.4
Umbilical artery pH	7.29 ± 0.04	7.30 ± 0.03	7.30 ± 0.03	7.30 ± 0.04

Data was shown as Mean ± SD, median (interquartile range) and number (%) as appropriate. *Adjusted *P* < 0.05, compared with dexmedetomidine groups.

**FIGURE 2:**
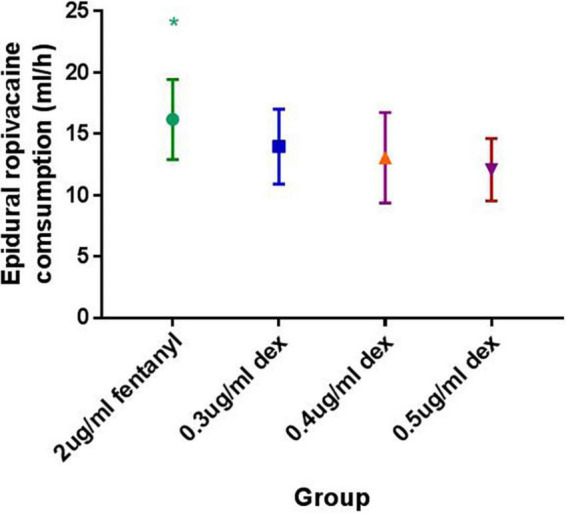
Mean hourly ropivacaine consumption between groups. *P < 0.0001 for one way ANOVA.

**FIGURE 3:**
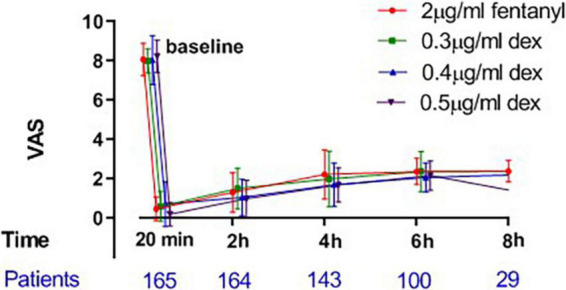
Mean VAS (0–10) ± SD over time during labor.

The mean hourly consumption of epidural ropivacaine was 16.2 ± 3.3, 14.0 ± 3.1, 13.1 ± 3.7 and 12.1 ± 2.5 ml/h in the 2 μg/ml fentanyl group, and the 0.3, 0.4, and 0.5 μg/ml dexmedetomidine groups, respectively. There was a significant difference among groups in the mean hourly consumption of epidural ropivacaine (*P* < 0.0001 for 1 way ANOVA, Figure 2). Tukey’s multiple comparisons test for mean hourly consumption of epidural ropivacaine showed there was a significant difference between the 2 μg/ml fentanyl group and the other three dexmedetomidine groups (adjusted *P* = 0.0205, 0.0002, and < 0.0001, respectively); no significant difference existed among dexmedetomidine groups. Totals of 82.5% (33/40), 44.6% (17/39), 44.4% (20/45) and 22.0% (9/41) of patients in 2 μg/ml fentanyl, 0.3, 0.4, and 0.5 μg/ml dexmedetomidine group required additional PCEA. The frequency of PCEA boluses were 3 (1, 3), 0 (0, 2), 0.5 (0, 2) and 0 (0, 1) for 2 μg/ml fentanyl, 0.3, 0.4, and 0.5 μg/ml dexmedetomidine group; and there was significantly higher in the 2 μg/ml fentanyl group than in the other three dexmedetomidine groups (*P* < 0.001) and similar in the dexmedetomidine groups (Table 2). Totals of 37.5% (15/40), 17.9% (7/39), 15.6% (7/45) and 12.2% (5/41) of patients in 2 μg/ml fentanyl, 0.3, 0.4, and 0.5 μg/ml dexmedetomidine group suffered “breakthrough pain”; the incidence of “breakthrough pain” was significant higher in group 2 μg/ml fentanyl than in other groups, all adjusted *P* < 0.05. The mean value of VAS scores among groups was similar over time (*P* > 0.05, Figure 3). Pain scores showed relief 20 min following epidural injection in all four groups. Although patient satisfaction of pain relief was significantly different among groups (*P* = 0.002), the overall mean satisfaction score was > 4.0 (1–5). Patients in the fentanyl group had a mean satisfaction value of 4.2, whereas patients in the 0.3, 0.4, and 0.5 μg/ml dexmedetomidine groups had mean values of 4.8, 4.7, and 4.8, respectively.

**TABLE 2:** Side effects and required patient-controlled epidural analgesia of epidural 2 μg/ml fentanyl verse three different concentrations of dexmedetomidine.

	Fentanyl 2 μg/ml	Dexmedetomidine 0.3 μg/ml	Dexmedetomidine 0.4 μg/ml	Dexmedetomidine 0.5 μg/ml	*P* value
Sample size	40	39	45	41	–
Pruritus	7 (17.5)*	0 (0.0)	0 (0.0)	0 (0.0)	<0.001
Bromage score > 1	2 (5.0)	1 (2.6)	3 (6.7)	9 (22.0)	0.007
Hypotension	5 (12.5)	3 (7.6)	5 (11.1)	7 (17.1)	0.635
Maternal Bradycardia	0 (0.0)	0 (0.0)	0 (0.0)	0 (0.0)	–
Fetal bradycardia	2 (5.0)	5 (12.8)	3 (6.7)	2 (4.9)	0.483
Shivering	3 (7.5)	1 (2.6)	0 (0.0)	2 (4.9)	0.294
Severe Sedation	0 (0.0)	0 (0.0)	0 (0.0)	0 (0.0)	–
Nausea and vomiting	0 (0.0)	0 (0.0)	0 (0.0)	0 (0.0)	–
Patient required PCEA	33 (82.5)*	17 (44.6)	20 (44.4)	9 (22.0)	0.005
Frequency of PCEA boluses	3 (1, 3)*	0 (0, 2)	0.5 (0, 2)	0 (0, 2)	<0.001

Data was shown as number (%), median (interquartile range).

*Adjusted *P* < 0.05, compared with dexmedetomidine groups. Hypotension was defined as a decrement > 20% from baseline blood pressure, or an absolute value < 90 mm Hg. Bradycardia was defined as heart rate < 50 bpm. Ru-Ying Pang: R-YP Yao-Hua Shen: Y-HS.

The incidence of pruritus in the fentanyl group was 17.5%; in contrast, no patient experienced pruritus in any of the dexmedetomidine groups, *P* < 0.001. There was a significant difference in Bromage score among groups, *P* = 0.007. Three patients had a Bromage score of 2 and six patients had a Bromage score of 1 in the 0.5 μg/ml dexmedetomidine group; three patients had a Bromage score of 1 in the 0.4 μg/ml dexmedetomidine group; one patient in the 0.3 μg/ml dexmedetomidine group had a Bromage score of 1; 2 patients had a Bromage score of 1 in 2 μg/ml fentanyl group. There was no difference in the side effects of nausea and vomiting, shivering, severe sedation, and respiratory depression (Table 2).

## Discussion

The results of the present study demonstrated that 0.3, 0.4 and 0.5 μg/ml of adjuvant dexmedetomidine reduced the mean hourly requirement of epidural ropivacaine in the standard PIEB plus PCEA infusion protocol for labor analgesia when compared to the use of the traditional epidural dose of 2 μg/ml of fentanyl; and with a lower frequency of pruritus. In the subgroup analysis, the mean hourly consumption of epidural ropivacaine was similar among the three different doses of dexmedetomidine. Although a dose-dependent reduction in local anesthetic consumption was not found, 0.5 μg/ml of dexmedetomidine combined with 0.125% ropivacaine was associated with a high degree of motor block in this study. Therefore, while our results confirmed that epidural adjuvant dexmedetomidine could reduce the dose requirement of epidural local anesthetic agent compared to fentanyl, we would advocate that the concentration of dexmedetomidine not be greater than 0.4 μg/ml when combined with 0.125% of ropivacaine for epidural labor analgesia.

From these data, it would seem appropriate to consider the use of adjuvant dexmedetomidine as an alternative to fentanyl to reduce the dose consumption of local anesthetic agents and further minimize the associated side effects. Recently, an opioid-free strategy for pain relief has been widely advocated by anesthesiologists with the purpose of decreasing opioid related side effects, potential for addiction, and to promote Enhanced Recovery after Surgery (12, 13). Although this study substantiated the superiority of dexmedetomidine to fentanyl in decreasing epidural ropivacaine and with less pruritus, larger studies would be appropriate to further compare the advantages and disadvantages of the two adjuvants before dexmedetomidine could be routinely preferred in clinical practice.

The exact mechanism of dexmedetomidine in reducing dose consumption of epidural local anesthetic remains unknown. It may exert its analgesic effect via the role of α2-AR adrenoceptors (2), via vasoconstriction (14), or through synergistic effects (15) with sodium channel blockers.

We chose fentanyl 2 μg/ml in this study because this concentration has been widely accepted clinically, and it has been well documented to reduce the dose requirement of epidural local anesthetic during labor analgesia (16). Moreover, epidural use of ropivacaine alone for labor analgesia would seem certain to necessitate an increase in its concentration, which would enhance lower limb motor block and reduce patient satisfaction. In fact, in the present study, the incidence of motor block in the 0.5 μg/ml of dexmedetomidine group was higher than in other lower-dose groups, implies that increasing doses of dexmedetomidine in the epidural solution could bring an increase in motor block. This perhaps explanation for this phenomenon may be due to a synergistic effect of dexmedetomidine with local anesthetics (15), through which dexmedetomidine not only enhanced the analgesic effect of local anesthesia, but also increased the side effect of motor block. However, this finding is the secondary outcome of this study, and the sample size may be not sufficient for this context and the possibility of statistical error cannot be excluded.

Of note, in a prior study but inconsistent with the results of our current study, we found that the use of epidural dexmedetomidine provided a dose-dependent reduction in the median effective concentration of ropivacaine for the induction of epidural labor analgesia (6). The explanation may be that using 0.125% of ropivacaine with 0.3 μg/ml dexmedetomidine for labor pain in conjunction with the PIEB plus PCEA protocol in both study designs may have caused patients to reach a plateau phase of relieving labor pain. If so, further increases in the dose of dexmedetomidine might only have resulted in non-therapeutic effects and even potentially increased risk of side effects such as a higher degree of motor block, as experienced in the 0.5 μg/ml dexmedetomidine group in the present study. Further study to determine the full dose-response of epidural ropivacaine with dexmedetomidine is warranted.

Although dexmedetomidine may be an important adjuvant alternative to opioids, especially in patients with extreme opioid sensitivity (vomiting and pruritus), the major disadvantage of this drug is the fact that it remains an investigational drug by the U.S. Food and Drug Administration for use in the epidural space. Similar to prior studies (6–8), our data showed no adverse effects of dexmedetomidine on maternal or neonatal outcomes. Human studies, as well as animal studies, have demonstrated the safety of using dexmedetomidine as a local adjuvant in peripheral, epidural, and intrathecal spaces without any neurological complications (17–19). Nevertheless, larger sample studies are warranted to further verify the safety of using dexmedetomidine as a neuraxial adjuvant.

There are limitations to the present study that need to be acknowledged. First, while the sample size was adequate to determine differences of our primary outcomes among the study groups, it was not powered sufficiently to definitively detect or reach conclusions on such aspects as side effects or other secondary outcomes. Second, due to the design of this study, the dose-response of dexmedetomidine on epidural ropivacaine was not clarified, and future studies on this topic may be of great interest. Third, although no additional adverse effects of dexmedetomidine were identified, there are no objective criteria for evaluating its neurological effects. Fourth, the objective of this study is to compare the mean amount of ropivacaine administered per hour among study groups, which was the primary outcome for which the study was powered. However, for the secondary outcomes, the sample size of the study may not be powered. Finally, patients experiencing “breakthrough pain” not rescued with a bolus of 10 ml of 0.25% ropivacaine plus 100 μg of fentanyl were excluded from the study, which could overestimate the effectiveness of the current analgesic strategy.

In conclusion, we found that epidural dexmedetomidine (0.3 and 0.4 μg/ml) is superior to epidural traditional fentanyl (2 μg/ml) in reducing hourly ropivacaine consumption and minimizing opioid-related side effects. Further large and multicenter studies are needed to validate adjuvant dexmedetomidine as an alternative to opioids before advising its routine clinical use.

## Data availability statement

The original contributions presented in the study are included in the article/supplementary material, further inquiries can be directed to the corresponding author.

## Ethics statement

The studies involving human participants were reviewed and approved by Ethical Committee of Lin-Ping District Women and Children Care Hospital, Hangzhou, China. The patients/participants provided their written informed consent to participate in this study.

## Author contributions

R-YP helped in designing and conducting the study, analyzing the data, and writing the manuscript. Y-HS, X-QJ, H-FX, YW, B-XZ, and S-FL helped in conducting the study and collecting the data. FX helped in the study design, data analysis, and manuscript preparation. All authors contributed to the article and approved the submitted version.
